# Thyroglossal Duct Cysts (TGDC) in the Elderly Population: The Role of Conservative Management

**DOI:** 10.7759/cureus.72183

**Published:** 2024-10-23

**Authors:** Maria K Pomponio, Keith R Conti, Jason F Ohlstein, Irfan Khan, Thomas Koch

**Affiliations:** 1 Otolaryngology - Head and Neck Surgery, St. Luke's University Health Network, Bethlehem, USA; 2 Otolaryngology - Head and Neck Surgery, Specialty Physician Associates, Bethlehem, USA; 3 Otolaryngology - Head and Neck Surgery, Lehigh Valley Health Network, Bethlehem, USA; 4 Pathology, St. Luke's University Health Network, Bethlehem, USA

**Keywords:** congenital neck mass, ectopic thyroid, elderly population, thyroglossal duct cysts, thyroid embryology

## Abstract

Sistrunk procedure is the treatment for thyroglossal duct cysts (TGDC), the majority of which occur in children. TGDC are very rare in the elderly and account for under 2% of cases. Management in this population, where risks of surgery may outweigh benefits, is unclear. Here we present two cases of elderly patients with asymptomatic to mildly symptomatic TGDC. Both patients were 87-year-old women who were found to have neck swelling on exam. They both underwent computed tomography (CT) scans and ultrasound-guided fine needle aspiration (FNA). Both had histology consistent with a thyroglossal duct cyst. The patients were managed conservatively without evidence of recurrence. We conclude that in elderly patients with FNA demonstrating TGDC and minimal to no symptoms, conservative management is reasonable.

## Introduction

Thyroglossal duct cysts (TGDC) are the most common congenital anomaly of the thyroid gland, the majority of which present in children or young adults [1]. Rarely, TGDC present in the elderly population [2]. Prior reports of TGDC in the elderly population have focused on severe presentations, including mass effect or concern for malignancy, which resulted in operative intervention [3-5]. The management of elderly patients with incidentally found TGDC has yet to be standardized. The standard of care for a TGDC identified in the pediatric population is operative excision via Sistrunk procedure, during which the TGDC and surrounding tissue are removed [6]. However, the elderly population presents unique considerations as the risks of surgery may outweigh the benefits. Due to the lack of published literature regarding conservative management of TGDC in the elderly population, we present two case reports of asymptomatic to mildly symptomatic TGDC who were treated conservatively. 

## Case presentation

Patient A

**Figure 1:**
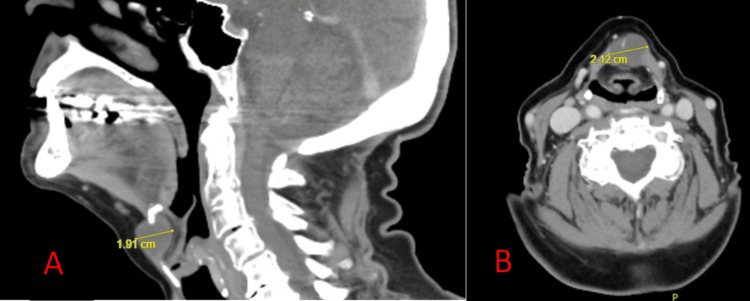
Computed Tomography (CT) Scan for Patient A Sagittal (A) and Axial (B) CT scan views for Patient A demonstrating a 1.9 cm x 1.9 cm x 2.1 cm lesion with septal calcifications embedded in the midline strap muscles with mild mass effect

The first patient is an 87-year-old female with a past medical history of hypertension, hyperlipidemia, and breast cancer status post mastectomy in 2008 who first presented to her primary care physician with a new-onset neck swelling for two days. The patient denied any recent upper respiratory symptoms. She did not have difficulty breathing, sore throat, or dysphagia. On physical exam, the Primary Care provider noticed a 3 cm non-tender, midline mobile mass. The area was not indurated or erythematous. The provider ordered a neck ultrasound that the patient had performed the same day. Her results demonstrated a 3.1 cm x 2.1 cm x 1.9 cm bi-lobar well-defined cystic lesion at the level of the hyoid bone. Two days later, she presented to a tertiary otolaryngology clinic. Physical examination by the Otolaryngologist revealed that the mass was mobile and fixed to the larynx at the level of the hyoid bone. The differential diagnosis is broad, and possibilities discussed with the patient included laryngeal carcinoma, lymph node, laryngocele, or TGDC. Cross-sectional imaging with a computed tomography (CT) scan was acquired due to risk of malignancy. This CT showed a 1.9 cm x 1.9 cm x 2.0 cm lesion with septal calcifications embedded in the midline strap muscles with mild mass effect (Figures 1A, 1B). 

**Figure 2:**
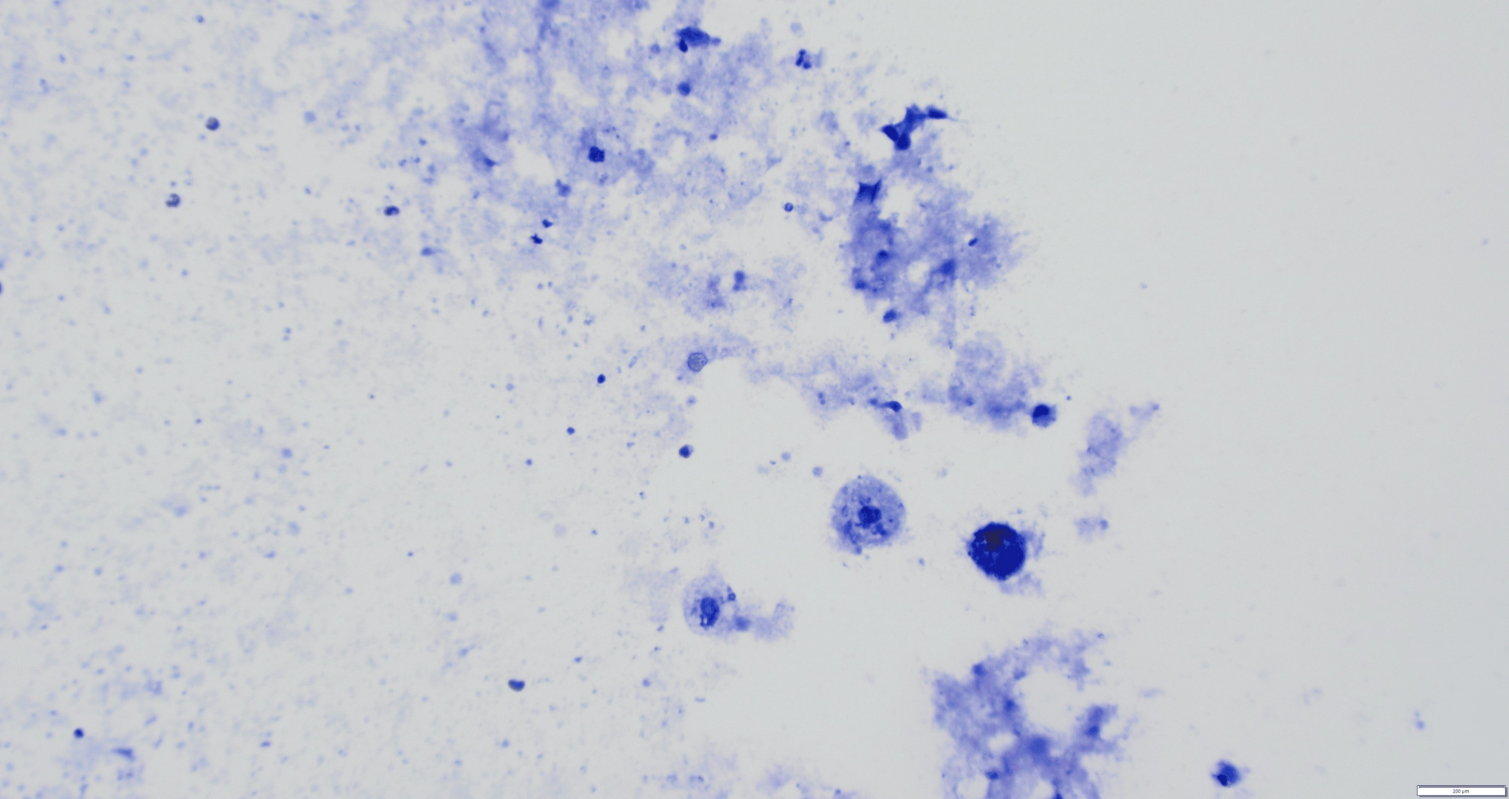
10x power view (medium-power view) of fine needle aspiration (FNA) slide showing thyroglossal duct cyst contents

The radiographic findings were consistent with a TGDC, but there was some concern for malignancy given the calcifications. The mass remained asymptomatic and stable in size when she returned for follow-up three months later. Treatment options discussed included watchful waiting, ultrasound-guided fine needle aspiration (FNA), or surgical excision with Sistrunk. The patient underwent ultrasound-guided FNA which was performed by the Radiology Department. Histopathologic analysis of the FNA specimen revealed benign ciliated columnar cells with colloid and macrophages, confirming the diagnosis of TGDC (Figure 2). Using a shared decision-making model, the patient elected not to pursue a Sistrunk procedure. She was contacted to get a repeat ultrasound in one year, but did not complete this. 

Patient B

**Figure 3:**
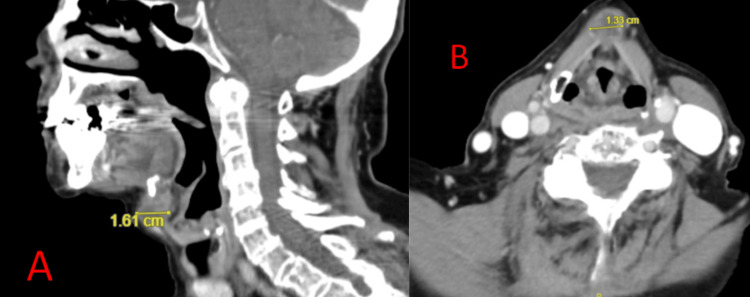
Computed Tomography (CT) Scan for Patient B Sagittal (A) and Axial (B) views from CT scan for Patient B demonstrating a midline, irregularly-shaped, cystic 1.3 cm x 1.6 cm x 2.0 cm mass just below the hyoid bone embedded within the strap muscles

The second patient is also an 87-year-old female with a past medical history of pre-diabetes, hypertension, hyperlipidemia, and hypothyroidism controlled on levothyroxine 88 mcg. She initially presented to a tertiary otolaryngology clinic with ear itching. During this visit, no palpable neck mass was identified. Ten months later, she represented to otolaryngology clinic with a one-month history of neck swelling. The patient first noticed midline anterior neck swelling following an upper respiratory tract infection. The patient reported a new onset lump in throat sensation. She denied sharp neck pain, difficulty breathing, or difficulty swallowing. Given the patient's new onset of globus sensation, flexible laryngoscopy was performed. The results of flexible laryngoscopy were normal and unrevealing of causes of globus sensation, such as pharyngeal mass or evidence of laryngopharyngeal reflux. However, physical examination revealed fullness in the hyoid region and palpable swelling. Pertinent negative include no lymphadenopathy, tenderness to palpation in the neck, or erythema to suggest active infection. The remainder of the physical exam was unremarkable. Given concern for a neck mass, a CT scan was obtained to rule out structural lesions as the cause of her symptoms. The CT revealed a midline, irregularly-shaped, cystic 1.3 cm x 1.6 cm x 2.0 cm mass just below the hyoid bone embedded within the strap muscles (Figures 3A, 3B). 

**Figure 4:**
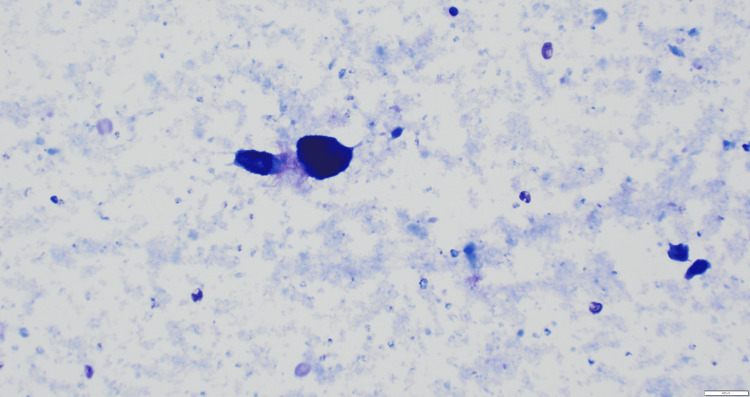
10x power view (medium-power view) of fine needle aspiration (FNA) slide showing dense colloid without evidence of hypercellularity or irregular nuclei to suggest malignancy, thus confirming the diagnosis of thyroglossal duct cyst (TGDC).

A thin connection was seen from the inferior portion of the mass to the right thyroid lobe. The radiographic findings were consistent with a TGDC. She returned to clinic one month later and the neck swelling and globus sensation had resolved. Despite the improvement in swelling, an ultrasound-guided FNA was recommended to rule out malignancy. The patient agreed to proceed with this procedure. The ultrasound-guided FNA was performed by the Radiology Department at our institution. Histopathologic analysis revealed macrophages, inflammatory cells and few epithelial cells, compatible with TGDC (Figure 4). She remains asymptomatic over two years later without recurrence of neck swelling. 

## Discussion

A TGDC is a failure of tract obliteration as the thyroid descends from the foramen cecum to its final position in the anterior neck [1,7]. As a consequence of embryonic failure, these are typically identified in the pediatric population [1]. 

Few case reports have demonstrated TGDC presenting in the elderly population [3-5,8,9]. Approximately 2% of cases occur after the sixth decade of life [2,9]. During review of the literature, it was found that all prior case reports focus on very severe presentations of either a large amount of anterior neck swelling or concern for malignancy [3,5,8]. In the majority of case reports of patients over the age of 60, the Sistrunk procedure was performed [3,5,8]. El-Ayman et al. documented a case of a large TGDC in an 85-year-old gentleman [3]. In this patient, the swelling first appeared as a young man and gradually progressed throughout his life. The patient had significant cosmetic impairment and trouble swallowing. Surgery was pursued due to the extent of the disease and final pathology was benign [3]. In another case report, Sudharsanan et al. described a case where a 76-year-old male presented with anterior neck swelling that had been present for three months [5]. The mass was firm on physical examination and a CT scan was obtained which showed a 5 cm x 4.5 cm cystic lesion in the hyoid region with septations [5]. An FNA was performed and revealed atypia of undetermined significance [5]. Due to concern for malignancy, the patient was taken for operative intervention. Final pathology was consistent with papillary thyroid cancer (PTC) [5]. 

Prior studies have indicated that TGDC should be removed in elderly patients due to a potentially higher rate of PTC [3]. The decision to proceed with operative intervention should be a thoughtful patient discussion addressing the patient’s symptoms and concerns within the broader context of their comorbidities and functional status. In patients reported on previously, such as the case reports discussed above, surgery was a reasonable option given concern for malignancy or significant functional impairment. The treatment choice in asymptomatic cases or minimally symptomatic cases remains unclear. Modern management of thyroid nodules includes ultrasound or CT followed by FNA with subsequent Bethesda scoring and genetic testing. No such guideline exists for TGDC. The rate of utilizing FNA in the diagnosis of adults with TGDC has been low [10,11]. In a large study including over 400 adults with TGDC, only 144 underwent FNA [10]. In another study, only 57% of patients with TGDC underwent FNA [11]. For most, FNA is well tolerated and a reasonable step before surgical intervention in adults with TGDC when combined with imaging modalities to exclude carcinoma. 

The situation becomes unclear if FNA is non-diagnostic or positive for PTC. The rate of non-diagnostic samples varied largely between the two reports, with non-diagnostic rates of 8 and 85% [10,11]. In the Thompson study, it was found that 85% of FNA samples on TGDC were non-diagnostic or unsatisfactory for evaluation [10]. The study does not postulate as to why their non-diagnostic rate is so high. It also does not specify if ultrasound guidance was used during FNA, which increases the rate of satisfactory samples. In contrast, Wong et al. reported that only 8% of FNA samples were unsatisfactory for evaluation [11]. This study used ultrasound guidance during all FNAs, which may explain the disparity in prior reports. 

For patients with FNA-proven PTC of their TGDC, conservative management still may be warranted. It is known that the overall survival rate of PTC is 99% at 20 years [12]. A SEER database study with over 50,000 patients found that the 20-year thyroid disease-specific survival (DSS) was excellent. The highest-risk population included, those with tumors 17-20 mm and nodal stage one disease, still had over a 95% thyroid cancer DSS at 20 years. This study concluded that active surveillance is an option for the majority of patients with papillary thyroid cancer [13]. In recent years, there has been more data recommending conservative approach for small, low-risk PTC [13]. In the elderly, the odds of mortality due to other causes may be greater than their mortality risk from a small PTC. Future research is needed to determine whether TGDC positive for PTC can be managed conservatively. 

## Conclusions

In conclusion, TGDC are very rare in patients above 65 years old. Management in this population, where risks of surgery may outweigh benefits, is not standardized. In patients with compressive symptoms and large malignancies, surgery is likely indicated. However, in patients with minimal symptoms, benign FNA, or low-grade PTC, conservative management can be considered. We recommend all patients be followed by their otolaryngologist. Ultrasound-guided FNA is indicated to exclude malignancy and confirm the diagnosis. It is unknown, however, whether repeat FNAs or imaging is needed in this population if symptoms remain minimal. Repeating an ultrasound is a low-cost option that could be considered in order to confirm stability of the TGDC. Further research is needed to create a standardized pathway and optimize patient care. In conclusion, we report two patients in their eighth decade of life with small TGDC that were successfully managed conservatively. 
